# The effect of antibiotics on protein diffusion in the *Escherichia coli* cytoplasmic membrane

**DOI:** 10.1371/journal.pone.0185810

**Published:** 2017-10-04

**Authors:** George S. Liu, Benjamin P. Bratton, Zemer Gitai, Joshua W. Shaevitz

**Affiliations:** 1 Department of Physics, Princeton University, Princeton, NJ, United States of America; 2 Lewis-Sigler Institute for Integrative Genomics, Princeton University, Princeton, NJ, United States of America; 3 Department of Molecular Biology, Princeton University, Princeton, NJ, United States of America; Centre National de la Recherche Scientifique, Aix-Marseille Université, FRANCE

## Abstract

Accumulating evidence suggests that molecular motors contribute to the apparent diffusion of molecules in cells. However, current literature lacks evidence for an active process that drives diffusive-like motion in the bacterial membrane. One possible mechanism is cell wall synthesis, which involves the movement of protein complexes in the cell membrane circumferentially around the cell envelope and may generate currents in the lipid bilayer that advectively transport other transmembrane proteins. We test this hypothesis in *Escherichia coli* using drug treatments that slow cell wall synthesis and measure their effect on the diffusion of the transmembrane protein mannitol permease using fluorescence recovery after photobleaching. We found no clear decrease in diffusion in response to vancomycin and no decrease in response to mecillinam treatment. These results suggest that cell wall synthesis is not an active contributor to mobility in the cytoplasmic membrane.

## Introduction

Intracellular transport is critical in cellular physiology. In eukaryotic and prokaryotic cells, motor and cytoskeletal proteins can enhance the active motion of particles. In turn, active motion can influence the diffusion of nearby passive particles through hydrodynamics or elastic interactions. Such “active diffusion” involves greater motion than can be explained by ordinary Brownian motion (i.e. passive diffusion), and has been observed in chromosomes in bacteria and yeast [1] and actin networks in eukaryotic cells [2–5]. Active diffusion also has been implicated in a broad range of cellular processes including regulation of the fluidic state of the cytoplasm [6], transcription of DNA [1,7], distribution of organelles [8], and metabolic alterations in malignancy [9]. Therefore, active diffusion may be a general phenomenon with wide-ranging consequences for cellular homeostasis.

Despite growing evidence that active motion can influence diffusion of nearby passive particles, few specific drivers of active diffusion have been identified. One candidate driver is cell wall synthesis. In bacteria, the cell wall is constantly being remodeled by penicillin-binding proteins (PBPs), which perform transpeptidase and glycosyl transferase reactions that are required for the processive motion of the bacterial actin homologue MreB in the cytoplasm [10–13]. Though the mechanical influence of cell wall synthesis does not appear to reach chromosomes in the prokaryotic nucleoid [1], its influence could reach particles in the cytoplasmic membrane where PBPs associated with cell wall synthesis are located. Previous work using vancomycin treatment to inhibit MreB motion suggests that active movement of MreB, driven by cell wall synthesis activity, could influence the diffusion dynamics of membrane proteins FruA and F_1_F_O_ ATP synthase [14]. We sought to further study the potential interactions between cell wall synthesis activity and the diffusion of membrane proteins using multiple antibiotic treatments targeting different steps of cell wall synthesis.

Here we investigate the contribution of cell wall synthesis to active diffusion in the cytoplasmic membrane of bacteria. We used fluorescent recovery after photobleaching (FRAP) to measure the apparent diffusion of mannitol permease (MtlA), a transmembrane protein not thought to be directly associated with the cell wall synthesis machinery [13], in individual *Escherichia coli* cells with and without antibiotic treatment targeting cell wall synthesis. We found that depletion of ATP with the proton ionophore carbonyl cyanide m-chlorophenylhydrazone (CCCP) decreased the apparent diffusion of MtlA in the cytoplasmic membrane. However, we did not find clear evidence of this effect for vancomycin, which inhibits and slows cell wall synthesis by binding peptide precursors of the cell wall. Blocking and slowing cell wall synthesis with mecillinam, which inhibits transpeptidation, did not appear to reduce MtlA diffusion. Our results suggest that cell wall synthesis motion does not appear to contribute to active diffusion in the *E*. *coli* cytoplasmic membrane.

## Materials and methods

### Bacterial strains

*Escherichia coli* strain VS116 (Δ*flhC* derived from the K12 strain RP437 [15]) was used for FRAP experiments without vancomycin treatment. A porous membrane *E*. *coli* strain with the *imp4213* mutation, derived from FB83 background [16], was used for vancomycin experiments. To express fluorescent transmembrane proteins, we used plasmid pVS266 which contains genes for ampicillin (AMP) resistance and yellow fluorescent protein (YFP) fused to mannitol permease A (MtlA) under isopropyl β-D-1-thiogalactopyranoside (IPTG) induction [15,17]. Plasmid pVS266 was isolated from the VS116 strain using the Qiagen mini-prep kit (QIAGEN, Hilden, Germany) and assessed for purity by UV spectroscopy. Transformation of *imp4213* mutants with the plasmid was performed by electroporation, following which transformed cells were grown on carbenicillin selective media overnight, and single colonies of resistant cells were picked, restreaked, and made into freezer stocks in 50% glycerol mixture of Luria-Bertani (LB) media.

### Sample preparation

Overnight cultures were grown in LB rich medium at 37°C. Daily cultures were made by diluting overnight cultures in M63 glucose with casamino acids (CA) minimal medium containing 20 mM IPTG [17]. Daily cultures were grown at 37°C with shaking to an optical density (OD_700_) of 0.3–0.5, then kept at room temperature and used for sample preparation for up to 3.5 hours. Samples were prepared by inoculating 1 μL of the daily culture onto an agarose pad on a glass slide. The slide was then covered with a coverslip, sealed with wax, and imaged for up to 45 minutes. Agarose pads were made by melting 300 μL of 2% agarose in water and mixing with 300 μL of 2x M63 glucose CA. The agarose pad solution was allowed to solidify at room temperature on the glass slide to a height of about three coverslips’ thickness. Antibiotic drugs were added at the desired concentration to the agarose pad before it solidified. Drug concentrations used in the agarose pad were 10 μg/mL CCCP, 100 μg/mL vancomycin, 100 μg/mL mecillinam, 100 μg/mL A22, and 100 μg/mL cefsulodin. These concentrations are above the minimum inhibitory concentration for blocking MreB rotation for the respective drugs [10].

### FRAP experiments

**Fig 1:**
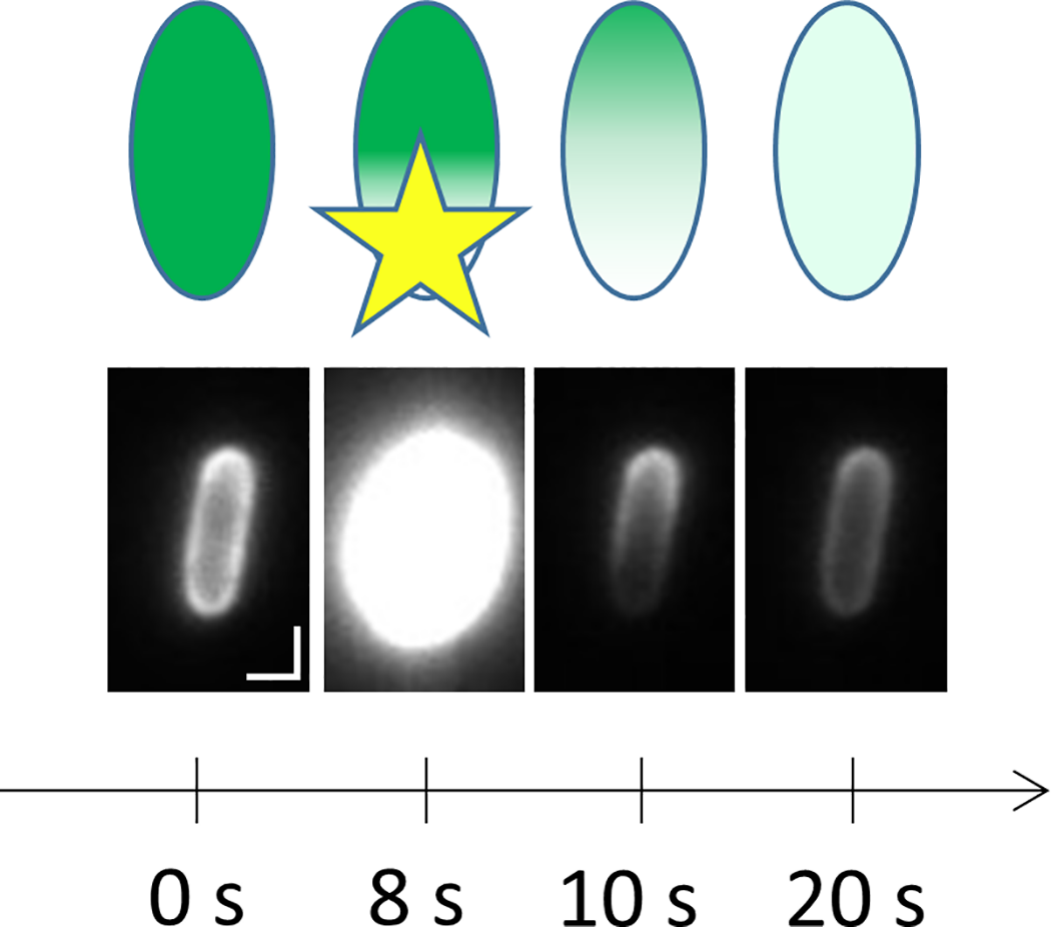
FRAP image acquisition. The schematic at the top shows the fluorescence intensity (*green shaded area*) changes in a fluorescent cell during the course of a FRAP experiment. The cell is bleached at 8 s at the bottom pole by the bleach beam (*yellow star*), and exhibits fluorescence recovery dynamics afterwards as shown at 10 s and 20 s. Corresponding bright-field images are shown below. In this example, the *E*. *coli* cells contain the MtlA-YFP membrane fluorescence-protein fusion (strain VS116::MtlA-YFP). Notice that the cell's outline is brighter than at its center, indicating the membrane localization of the fluorescent protein. During bleaching at 8 s, the bleach intensity is so high that photons spill over into the other acquisition channels of the CCD camera, creating the illusion of fluorescence beyond the cell borders. Scale bar 1 μm.

The custom-built microscope for bright-field and fluorescent imaging was described previously [18]. For photobleaching experiments, custom software written in LabVIEW (National Instruments, Austin, TX) was used to flip a lens to focus the 488 nm excitation laser to a diameter of about 0.9 μm to bleach YFP in the region of interest (ROI), usually a pole of the cell. Fluorescence images were recorded before and after bleaching with a CCD camera filtered for the emission photon wavelength at an acquisition rate of 2 s, laser power of 5 mW, and bleach duration of 1.5 s. Cells with uniform-appearing fluorescent intensity were chosen for bleaching. Representative schematic and fluorescent images are shown in Fig 1.

### Image analysis

**Fig 2:**
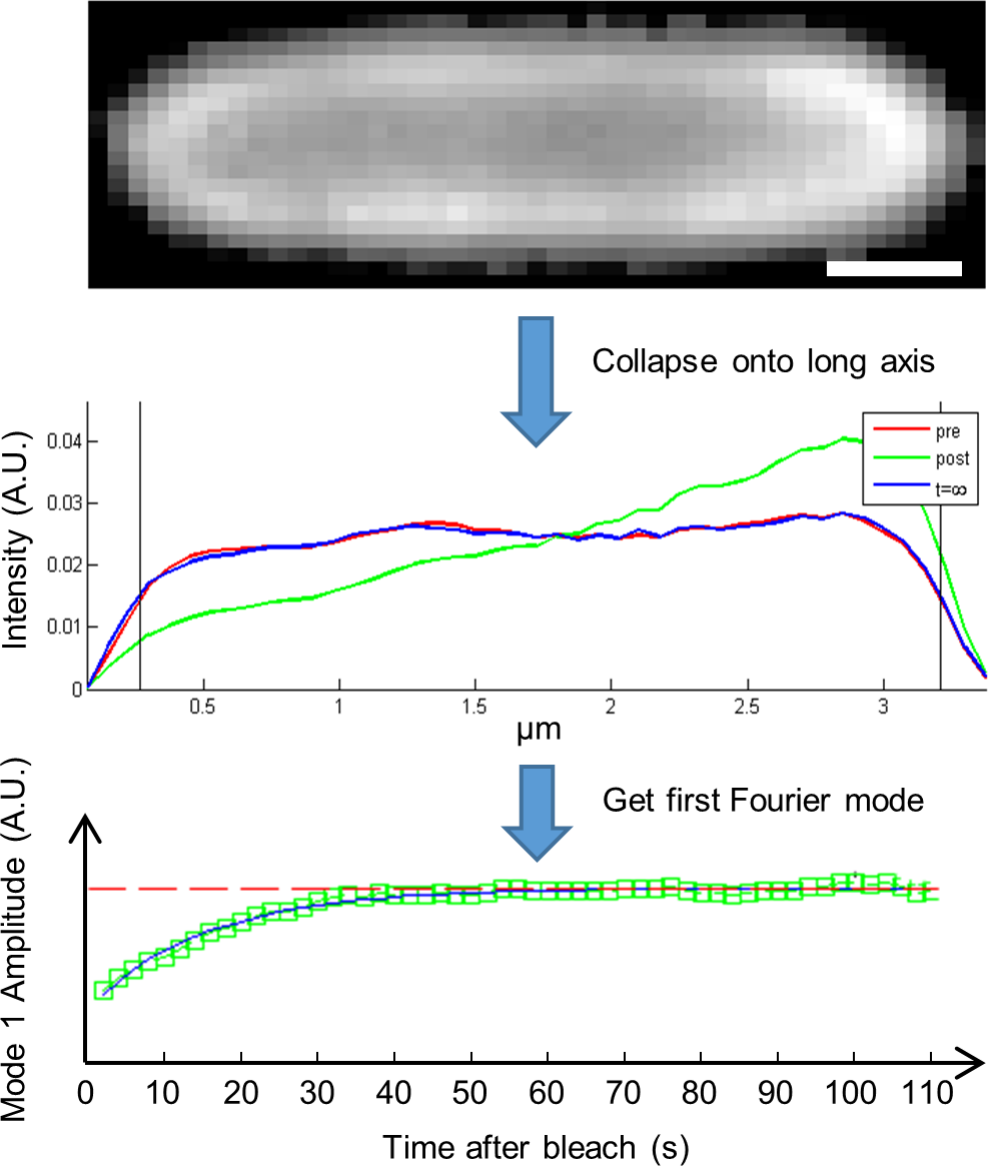
Extraction of a one-dimensional intensity profile from a two-dimensional fluorescent image. A two-dimensional fluorescent image of an *E*. *coli* cell expressing the MtlA-YFP fluorescent fusion protein is shown at the top. The image has been rotated to make the cell’s long-axis horizontal. The one-dimensional intensity profile (arbitrary units; A.U.) for the image is shown in the middle. It is scaled to match the length of the cell. The intensity profile is calculated using the average intensity of each vertical stripe (column) of pixels in the image at each position along the cell’s long axis. Black vertical lines indicate the calculated ends of the cell, where the intensity profile is at half of its maximum value. In each frame, the intensity profile was normalized to eliminate effects of uniform photobleaching over the course of imaging. This results in similar, uniform intensity profiles in the first pre-bleach (*red curve*) and last post-bleach (*blue curve*) intensity profiles. The intensity profile immediately after photo-bleaching (*green curve*) shows reduced fluorescence at the left pole, where the cell was photobleached. The plot at the bottom shows the calculated amplitudes of the first (non-constant) Fourier cosine modes of intensity profiles after photobleaching (*green squares*). The fit of these data to an exponential decay function (*black curve*) is used to estimate the apparent diffusion coefficient of the fluorescent protein. The post-bleach values of first Fourier cosine mode amplitude approach that of images before photobleaching (*red dashed horizontal line*). Pre, pre-bleach; post, post-bleach; t = ∞, last image in the time series. Scale bar 0.5 μm.

Experimental data consisted of time sequences of fluorescent recovery images of *E*. *coli* cells. Prior to analysis, each time sequence of images was visually inspected to ensure the presence of fluorescent photo-bleaching and recovery in the cell, and each frame was normalized for intensity to account for gradual photo-bleaching. In each frame, the cell was segmented by thresholding using Otsu’s method [19]. If multiple cells were present in the field of view, the coordinate of the bleach spot was used to identify the photo-bleached cell. The two-dimensional image of the cell was converted to a one-dimensional fluorescence intensity profile by fitting an ellipse with the same second moment as the cell to determine the cell axis, grouping pixels in stripes perpendicular to the cell axis, and squashing each stripe to a single value equal to the average intensity of the pixels in the stripe. Using a frame of the cell before photo-bleaching, the ends of the cell were determined as the positions where the one-dimensional intensity profile was at half of its maximum value. Consequently, the cell length was measured as the full width at half maximum of the one-dimensional intensity profile before photo-bleaching. Only the region between the ends of the cell were used for further analysis of the one-dimensional intensity profile. An example of the intensity profile of a cell and its calculated ends are shown in Fig 2.

The coefficient of apparent diffusion of YFP-MtlA was determined by measuring the single-exponential decay time of the one-dimensional intensity profile as previously described [20–22]. Briefly, our analysis is based on the solution to the one-dimensional form of Fick’s second law of diffusion $\frac{\partial I(x,t)}{\partial t}=D\frac{\partial^{2}I(x,t)}{\partial x^{2}}$, with boundary conditions ${\frac{\partial I(x,t)}{\partial x}|}_{x=0}={\frac{\partial I(x,t)}{\partial x}|}_{x=L}=0$. Here, *I*(*x*,*t*) is the one-dimensional intensity profile of the cell, *D* is the coefficient of apparent diffusion, and *x* = 0 and *x* = *L* are the positions of the cell ends in the coordinate system where the cell axis lies on the x-axis. The general solution is the Fourier cosine series:

$$I(x,t)=\sum_{n=0}^{\infty }A_{n}(t)\cos\left(\frac{n\pi }{L}x\right)$$ (1)

where *A*_*n*_(*t*) is the amplitude of the n-th Fourier cosine mode (i.e. mode *n*), and is calculated from *I*(*x*,*t*) using the formula:

$$A_{n}(t)=\frac{2}{L}\int_{0}^{L}I(x,t)\cos\left(\frac{n\pi }{L}\right)dx$$ (2)

The amplitude of each Fourier cosine mode decays exponentially with time, independent of the other modes, according to the formula $A_{n}(t)=A_{n}(0)e^{-{\left(\frac{n\pi }{L}\right)}^{2}Dt}$, for cells bleached at (or before) *t* = 0. We calculated the amplitude of the Fourier cosine mode in each frame of the time-sequence of images, using the one-dimensional intensity profile as described in Eq (1), and fit the data to a single-exponential decay function *A*_*fit*_(*t*) = *ae*^−*bt*^ + *c*. We then used the fitted parameters to calculate the coefficient of apparent diffusion $D=\frac{bL^{2}}{n^{2}\pi^{2}}$. This calculation effectively translates the time constant $\tau =\frac{1}{b}=\frac{L^{2}}{n^{2}\pi^{2}D}$ measured from the relaxation of the *n*th Fourier cosine moment to the diffusion coefficient *D*, and is calculated using the fitted parameter *b* (which is the reciprocal of the time constant *τ*), the known quantity *n*, and the measured cell length *L*. In this work, we measured the first Fourier cosine mode (i.e. mode *n* = 1) because it decays slow enough to be tracked at our camera’s acquisition rate [20–22].

We note that the calculation of the apparent diffusion coefficient (*D*) depends on cell size in the direction along which diffusion is being measured (in our case, the relevant cell size is length (*L*) along the cell’s long axis). Notably, cell size in the other dimensions does not play a role in our calculations of diffusion coefficients because passive diffusion involves a random walk in which steps in x, y, and z are independent, and hence diffusive motion along the cell’s long axis is independent of motion (and cell size) in the other dimensions. Because cell length *L* is measured in each cell to calculate the apparent diffusion coefficient *D*, diffusion measurements can be compared across different samples even when the morphology of cells is not constant across samples.

The key assumption for our diffusion measurements is the continuity equation $\frac{\partial \rho }{\partial t}=-\nabla \cdot j$, where *ρ* is the concentration and **j** is the flux of fluorescent proteins. This is the case when changes in concentrations of fluorescent proteins are due to diffusion rather than synthesis or destruction of fluorescent proteins. This assumption is likely satisfied in our experiments because our fluorescent recovery dynamics occur on the timescale of seconds (Fig 2). By contrast, the typical bacterial protein half-life is several hours [23], and fluorescent protein synthesis occurs on the timescale of minutes; prokaryotic translation takes about 20 amino acids per second with additional time for folding [24] and MtlA-YFP has a molecular mass of is 189.3 kDa [17], equivalent to about 1720 amino acids (since the average molecular weight of an amino acid is 110 Da). Fluorescent proteins do not leave or enter the cell at its edges because the cell membrane is bound; net flux of protein into or out of the cell would mimic the effects of protein synthesis or destruction, respectively. Our description of measurements as apparent diffusion coefficients further acknowledges the possibility that viscoelastic effects and other non-diffusive motions may play a role in affecting our diffusion coefficient measurements.

During FRAP experiments, the bleach spot was placed near the pole of the cell to maximize the initial amplitude of the first Fourier cosine mode (Fig 1). An overview of the FRAP analysis procedure is shown in Fig 2. Computational analysis of image data was performed using custom code in MATLAB (The Mathworks, Natick, Massachusetts).

### Statistical analysis

Statistics were performed in MATLAB. P-values were calculated using the unpaired Student’s t-test. P-values are reported but were not used to determine similarities or differences among data. All p-values and sample sizes are given in the figures.

## Results

### CCCP reduces the apparent diffusion of transmembrane protein MtlA in the *E*. *coli* cytoplasmic membrane

**Fig 3:**
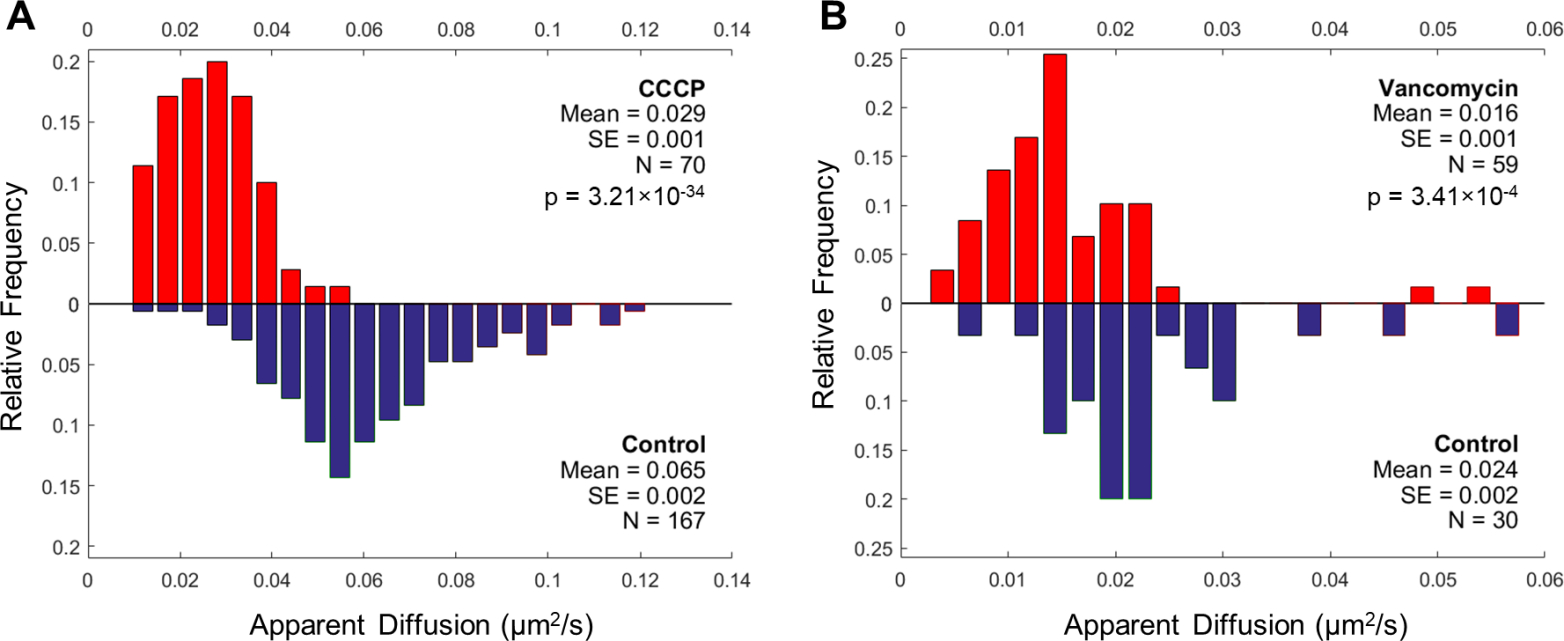
FRAP measurements of MtlA apparent diffusion in *E*. *coli* cells treated with CCCP and vancomycin. (A) The apparent diffusion of MtlA was measured in cells with (*red*) and without CCCP treatment (*blue*). (B) Apparent diffusion coefficient of MtlA in *E*. *coli* cells containing the *imp4213* mutation with (*red*) and without vancomycin treatment (*blue*).

We investigated whether treatment of *E*. *coli* with antibiotics could reduce the apparent diffusion of transmembrane protein MtlA in the cytoplasmic membrane. To test this hypothesis, we used FRAP to measure the diffusion of YFP-MtlA in *E*. *coli* cells growing in different antibiotic conditions. We first measured diffusion of YFP-MtlA in cells treated with carbonyl cyanide m-chlorophenylhydrazone (CCCP). CCCP exerts several non-specific effects in cells by depleting ATP through uncoupling of oxidative phosphorylation and ATP synthesis [25], which may interfere with active processes that enhance MtlA active diffusion. We observed a lower coefficient of apparent diffusion of MtlA-YFP in CCCP-treated cells compared with in untreated cells (0.029 ± 0.001 vs 0.065 ± 0.002 μm^2^/s, mean ± SE; unpaired t-test, p<0.001) (Fig 3A). These results support the notion that active motion may be coupled with passive motion of MtlA, and demonstrates an inhibitory effect of CCCP on motion of transmembrane proteins in *E*. *coli*.

### Vancomycin may reduce the apparent diffusion of transmembrane protein MtlA in the *E*. *coli* cytoplasmic membrane

To more specifically study the contribution of cell wall synthesis activity to membrane protein mobility, we measured MtlA diffusion in vancomycin-treated porous *E*. *coli* cells containing the *imp4213* mutation, which confers outer membrane permeability defects that increase susceptibility to vancomycin [26]. Vancomycin is a transpeptidation substrate inhibitor that inhibits cell wall synthesis and coupled MreB motion [10]. The apparent diffusion of MtlA appeared to be reduced in vancomycin-treated cells compared with controls (0.016 ± 0.001 vs 0.024 ± 0.002 μm^2^/s, mean ± SE; unpaired t-test, p<0.001) (Fig 3B). (We report the p-value for the Student unpaired t-test, but do not use it to determine significant differences for comparing or contrasting our experimental data.) This result suggests that vancomycin-targeted metabolic activity, such as cell wall synthesis activity, may increase motion of passive particles in the cytoplasmic membrane. However, because vancomycin treatment measurements were made in FB85 background *E*. *coli* containing the *imp4213* mutation, which involved a different strain compared with the K12 background *E*. *coli* used for CCCP measurements, it was difficult to compare the reduction in apparent diffusion of MtlA observed for vancomycin treatment with that observed for CCCP treatment, our positive control. Therefore, these results may not provide sufficient evidence to establish an inhibitory effect of vancomycin treatment on motion of transmembrane proteins in *E*. *coli*.

### Mecillinam, A22, and cefsulodin do not reduce the apparent diffusion of MtlA in the *E*. *coli* cytoplasmic membrane

**Fig 4:**
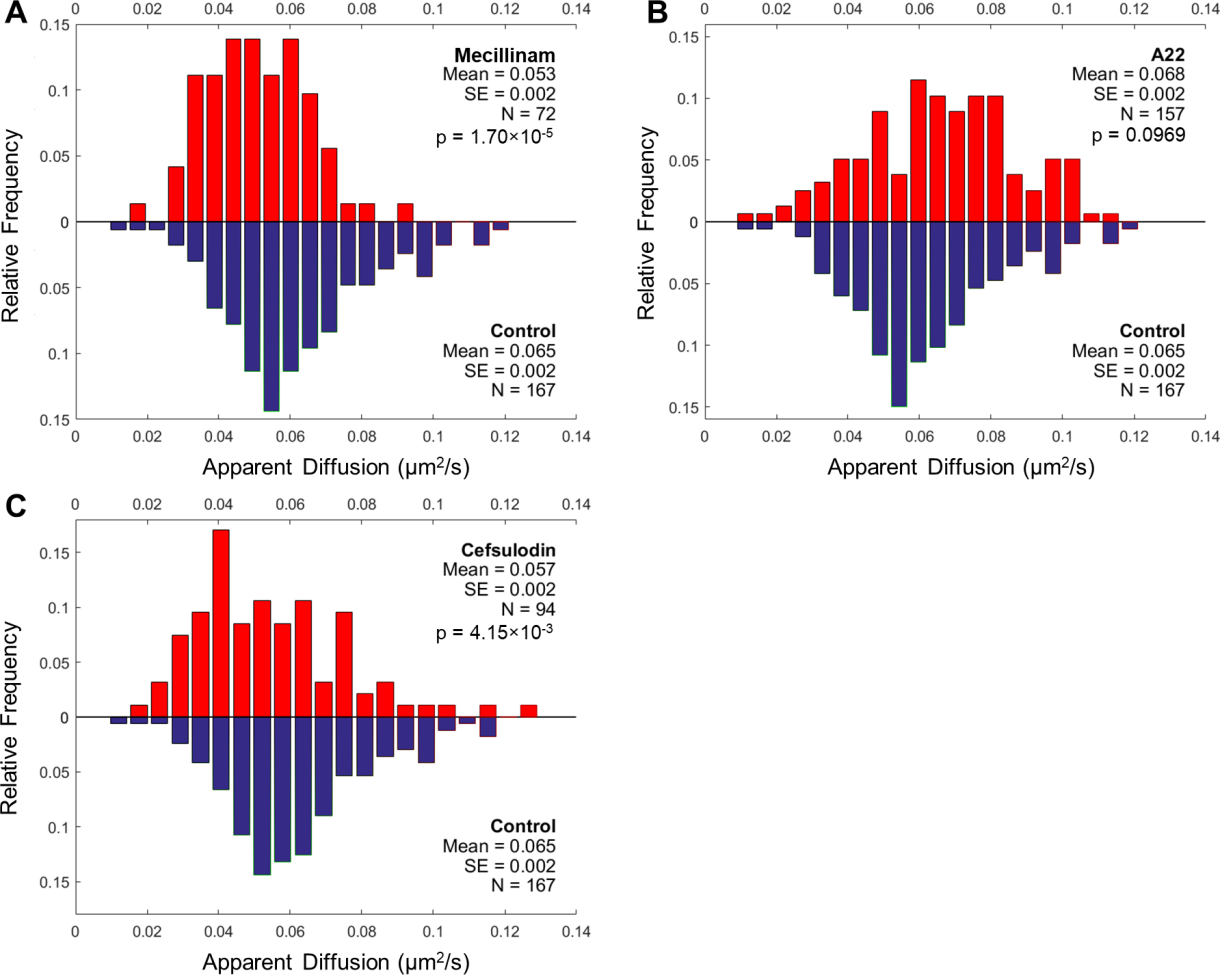
FRAP measurements of MtlA apparent diffusion in *E*. *coli* cells treated with mecillinam, A22, and cefsulodin. (A) The apparent diffusion coefficient of MtlA was measured in cells treated with (*red*) and without mecillinam treatment (*blue*). (B) Apparent diffusion coefficient of MtlA in cells with (*red*) and without A22 treatment (*blue*). (C) Apparent diffusion coefficient of MtlA in cells with (*red*) and without cefsulodin treatment (*blue*).

To further investigate the role of cell wall synthesis in influencing membrane protein mobility, we measured MtlA diffusion in *E*. *coli* cells treated with mecillinam. Mecillinam specifically targets penicillin-binding protein 2 (PBP2) [13] and reduces the movement of MreB in the cytoplasm [10]. We hypothesized that mecillinam treatment would inhibit MtlA diffusion in treated *E*. *coli* cells compared with in controls. However mecillinam treatment did not reduce the apparent diffusion of MtlA in treated cells compared with in untreated cells (0.053 ± 0.002 vs 0.065 ± 0.002 μm^2^/s, mean ± SE; unpaired t-test, p<0.001) (Fig 4A). Although the p-value for the t-test null hypothesis was small, histograms of the data showed no clear difference in the distributions of the apparent diffusion coefficients (Fig 4A). This result does not support a role for cell wall synthesis in facilitating active diffusion of MtlA in *E*. *coli*. However it leaves open the possibility that another active process that is unaffected by mecillinam, and affected by CCCP and perhaps vancomycin, may facilitate motion of MtlA.

As negative control experiments, we measured MtlA diffusion in *E*. *coli* treated with S-(3,4-dichlorobenzyl)isothiourea (A22) and cefsulodin. These drugs were not expected to reduce MtlA diffusion because they do not inhibit the motion of MreB coupled to cell wall synthesis [10]. A22 inhibits MreB polymerization [27] and cefsulodin is a third-generation cephalosporin that targets PBP1a/1b in *E*. *coli* [28,29]. We observed no reduction in MtlA diffusion in A22-treated cells compared with in controls (0.068 ± 0.002 vs 0.065 ± 0.002 μm^2^/s, mean ± SE; unpaired t-test, p = 0.097) (Fig 4B). We also observed no reduction in MtlA diffusion in cefsulodin-treated cells compared with in controls (0.057 ± 0.002 vs 0.065 ± 0.002 μm^2^/s, mean ± SE; unpaired t-test, p = 0.004) (Fig 4C). These results provide a baseline for interpreting associations between MtlA diffusion and antibiotic treatments.

## Discussion

Understanding how cells generate active diffusion is important for evaluating their metabolic and physical states [6,9,30]. With the exception of myosin-actin fluctuations [5,9,31,32], the identities of specific drivers of active diffusion are poorly understood. This is despite clear evidence that active diffusion is exhibited by cytoplasmic substrates and DNA [7,9]. Our investigation suggests that cell wall synthesis does not contribute to active diffusion of transmembrane proteins. In particular, mecillinam treatment did not reduce the coefficient of apparent of MtlA, and the effects of vancomycin are unclear.

This work relates to the existing literature in several ways. First, our finding that vancomycin treatment may reduce the apparent diffusion of transmembrane proteins may support previous observations of this effect [14]. Second, our result that A22 does not reduce the globally-measured apparent diffusion of MtlA supplements previous observations by single molecule tracking that A22 treatment increases local mobility of Dil-C12 in *E*. *coli* [33], suggesting perhaps that MreB polymerization locally drives transmembrane protein motion. Finally, our measurements of MtlA diffusion in untreated *E*. *coli* cells roughly confirm previous measurements of MtlA diffusion [17] (0.0721 ± 0.0025 vs 0.0283 ± 0.0027 μm^2^/s, mean ± 95% CI). The different growth conditions used to prepare daily cultures in our experiments may partially explain some of the difference between our diffusion measurements and previously reported values [17].

An estimate of the plausibility of active diffusion existing in the cytoplasmic membrane can be calculated using the Péclet number for MtlA, which compares its rates of advection and diffusion [34]. The formula for the Péclet number is $Pe=\frac{lv}{D}$, where Pe is the Péclet number, *l* is the distance for transport, *v* is the advective transport speed, and *D* is the diffusion coefficient. A Pe > 1 suggests that mixing through hydrodynamics may be effective. Using MreB velocity as a proxy for cell wall synthesis velocity, a generous estimate of the mixing speed in the *E*. *coli* plasma membrane is 10 nm/s [10]. For the membrane protein MtlA, which diffuses at a rate of about 0.06 μm^2^/s, the Péclet number for travel across a distance of 1 μm, the approximate length of an *E*. *coli* cell, is Pe = 1/6. This value suggests that advective transport could play a role in active diffusion of MtlA.

In summary, our data does not support a role for cell wall synthesis in enhancing the diffusion of transmembrane proteins in *E*. *coli* cytoplasmic membrane. Future work could investigate the biophysical mechanism by which vancomycin treatment may reduce the apparent diffusion of MtlA in the cytoplasmic membrane. Drugs such as ramoplanin and fosfomycin, and the *E*. *coli asd-1* mutant which possesses a genetic defect in synthesizing an essential component of peptidoglycan [35], could also be used to assess MtlA motion as they provide alternative conditions in which MreB motion is reduced in the *E*. *coli* cytoplasmic membrane [10].

## Supporting information

S1 Dataset Apparent diffusion coefficients of MtlA measured by FRAP in *E. coli* cells under different antibiotic conditions. (XLSX)
